# Predicting severe or critical symptoms in hospitalized patients with COVID-19 from Yichang, China

**DOI:** 10.18632/aging.202261

**Published:** 2020-12-09

**Authors:** Xin Chen, Feng Peng, Xiaoni Zhou, Jiang Zhu, Xin Chen, Yingying Gong, Wang Shupeng, Wenquan Niu

**Affiliations:** 1 Department of Cardiology, The First Affiliated Hospital of Fujian Medical University, Fuzhou, Fujian, China; 2 Department of Pulmonary Disease, The Third People’s Hospital of Yichang, Yichang, Hubei, China; 3 Department of Cardiology Nursing Center, The First Affiliated Hospital of Fujian Medical University, Fuzhou, Fujian, China; 4 Institute of Clinical Medical Sciences, China-Japan Friendship Hospital, Beijing, China

**Keywords:** COVID-19, severe or critical symptoms, nomogram, prediction, risk

## Abstract

Objectives: We aimed to identify potential risk factors for severe or critical coronavirus disease 2019 (COVID-19) and establish a prediction model based on significant factors.

Methods: A total of 370 patients with COVID-19 were consecutively enrolled at The Third People’s Hospital of Yichang from January to March 2020. COVID-19 was diagnosed according to the COVID-19 diagnosis and treatment plan released by the National Health and Health Committee of China. Effect-size estimates are summarized as odds ratio (OR) and 95% confidence interval (CI).

Results: 326 patients were diagnosed with mild or ordinary COVID-19, and 44 with severe or critical COVID-19. After propensity score matching and statistical adjustment, eight factors were significantly associated with severe or critical COVID-19 (*p* <0.05) relative to mild or ordinary COVID-19. Due to strong pairwise correlations, only five factors, including diagnostic delay (OR, 95% CI, *p*: 1.08, 1.02 to 1.17, 0.048), albumin (0.82, 0.75 to 0.91, <0.001), lactate dehydrogenase (1.56, 1.14 to 2.13, 0.011), white blood cell (1.27, 1.08 to 1.50, 0.004), and neutrophil (1.40, 1.16 to 1.70, <0.001), were retained for model construction and performance assessment. The nomogram model based on the five factors had good prediction capability and accuracy (C-index: 90.6%).

Conclusions: Our findings provide evidence for the significant contribution of five independent factors to the risk of severe or critical COVID-19, and their prediction was reinforced in a nomogram model.

## INTRODUCTION

In late 2019, a novel human coronavirus, severe acute respiratory syndrome coronavirus 2 (SARS-CoV-2), was first identified in Wuhan, China. The syndrome of clinical symptoms caused by SARS-CoV-2 is labelled as coronavirus disease 2019 (COVID-19) [1]. In just over two months, COVID-19 had rapidly spread across the globe, and on 11 March 2020 it was declared a pandemic by the World Health Organization. As of July 29, 2020, there have been 16,558,289 confirmed cases of COVID-19, including 656,093 deaths across 216 countries, areas or territories (https://covid19.who.int/).

Clinically, COVID-19 can manifest on a spectrum of illnesses ranging from mild, self-limiting respiratory tract illness to severe progressive pneumonia, multi-organ failure, and death eventually [2, 3]. The major symptoms of COVID-19 include fever, cough, fatigue, myalgia or arthralgia, sore throat, headache, shortness of breath, and sputum production [4]. High hopes have been pinned on the anti-HIV drug lopinavir-ritonavir in the treatment of COVID-19, yet the results are not satisfactory [2, 5, 6]. While no therapeutics have yet been proven effective and the true pathogenetic mechanisms of COVID-19 are not fully understood, the identification of risk factors in predicting the occurrence of critical illness in patients with COVID-19 is crucial to developing prevention strategies. Currently, several prediction models for COVID-19 diagnosis and prognosis have been established [7–10], with no consensus on their implications. Hence, the identification and characterization of risk profiling for the onset and progression of COVID-19 symptoms is still subject to exploration, improvement, and renewal.

To fill this gap in knowledge and yield more information for future studies, we, in 370 patients with COVID-19 consecutively admitted to The Third People’s Hospital of Yichang, aimed to identify potential risk factors for severe or critical COVID-19 and establish a prediction nomogram model to facilitate clinical application.

## RESULTS

### Baseline characteristics

There were 326 patients diagnosed with mild or ordinary COVID-19, and 44 patients with severe or critical COVID-19. Of the 44 patients with severe or critical COVID-19, 16 were progressed from mild COVID-19 during hospitalization and 28 were initially diagnosed to have severe or critical COVID-19 at admission.

The baseline characteristics of these patients are provided in Supplementary Table 1. Of note, patients with severe or critical COVID-19 were significantly older than patients with mild or ordinary COVID-19 (*p* <0.001). Over 90% patients with severe or critical COVID-19 had contact with Wuhan, remarkably higher than that (54.5%) in patients with mild or ordinary COVID-19 (*p* <0.001). Additionally, patients with severe or critical COVID-19 were more likely to be complicated with hypertension, diabetes, cerebrovascular disease, and cardiovascular disease than patients with mild or ordinary COVID-19 (all *p* <0.01). In view of these remarkable differences in above demographic characteristics, to counterbalance these differences, a propensity score matching method was employed accordingly.

After matching on age, sex, smoking, hypertension, diabetes, cerebrovascular disease, and cardiovascular disease, 43 patients with severe or critical COVID-19 and 70 patients with mild or ordinary COVID-19 were retained for the following analyses, and their baseline characteristics are summarized in Table 1.

**Table 1:** The baseline characteristics of study patients with COVID-19 after propensity score matching.

**Characteristics**	**Mild or ordinary cases (n=70)**	**Severe or critical cases (n=43)**	***p***
Age (years)	66 (55, 73)	70 (58, 79)	0.089
Males	38 (54.3%)	23 (53.5%)	0.934
Contact with WH	20 (31.7%)	39 (90.7%)	<0.001
Death	0 (0%)	15 (34.9%)	<0.001
Hypertension	27 (38.6%)	21 (48.8%)	0.284
Diabetes	9 (12.9%)	10 (23.3%)	0.151
Smoking	11 (15.7%)	6 (14%)	0.799
Cerebrovascular disease	3 (4.3%)	5 (11.6%)	0.140
Cardiovascular disease	7 (10.0%)	9 (20.9%)	0.106
Diagnostic delay (days)	3.500 (2, 6)	6 (3, 10)	0.005
WBC	4.250 (3.400, 5.700)	5.500 (4.400, 7.800)	0.010
NEUT	65.30 (56.30, 70.70)	79.60 (65.60, 89.90)	<0.001
LYMPH	26.20 (17.50, 33.30)	13.70 (6.900, 22.90)	<0.001
MONO	7.100 (4.500, 8.800)	4.200 (2.500, 7.500)	0.002
NEUT (×10^9^/L)	2.770 (2.030, 3.980)	4.600 (2.860, 6.670)	0.001
LYMPH (×10^9^/L)	1.130 (0.780, 1.430)	0.760 (0.530, 1.030)	<0.001
MONO (×10^9^/L)	0.275 (0.200, 0.370)	0.280 (0.200, 0.360)	0.455
PLT (×10^9^/L)	123 (102, 173)	134 (88, 182)	0.795
PCT (ng/mL)	0.100 (0.0700, 0.140)	0.130 (0.0800, 0.270)	0.069
TBIL (μmol/L)	9.590 (7.310, 14.45)	8.890 (6.920, 14.36)	0.665
DBIL (μmol/L)	2.410 (1.790, 3.870)	3.050 (2.210, 4.960)	0.060
ALB (g/L)	37.10 (33.70, 41.20)	29.60 (27.40, 36.10)	<0.001
ALT (UL)	21 (16, 43)	23 (14, 35)	0.561
AST (UL)	25 (19, 33)	24 (21, 36)	0.651
Cr (μmol/L)	69.75 (57.60, 80.20)	65.70 (54.50, 86.30)	0.546
CK (UL)	66 (42, 106)	80 (45, 130.5)	0.485
CKMB (UL)	13.15 (10.50, 16.10)	15 (9.900, 26.20)	0.392
CRP (mg/L)	27.60 (7.100, 67.10)	40.10 (13.20, 66.30)	0.270
LDH (UL)	205 (171, 259)	314 (217, 451)	<0.001
HGB (g/L)	119 (109, 129)	114 (97, 122)	0.007

Abbreviations: WH, Wuhan; WBC, white blood cell; NEUT, neutrophil; LYMPH, lymphocyte; MONO, monocyte; PLT, platelet; PCT, procalcitonin; TBIL, total bilirubin; DBIL, direct bilirubin; ALB, albumin; ALT, alanine transaminase; AST, aspartate aminotransferase; Cr, creatinine; CK, creatine kinase; CKMB, creatine phosphokinase-isoenzyme-MB; CRP, C-reactive protein; LDH, lactate dehydrogenase; HGB, hemoglobin. The *p* value was calculated using the rank sum test or χ^2^ test where appropriate. Data are expressed as median (interquartile range) or count (percentage).

### Identification of significant factors for severe or critical COVID-19

As shown in Table 2, before adjustment, eight factors were in statistically significant association with the risk of severe or critical COVID-19 when compared with mild or ordinary COVID-19 (*p* <0.05), including diagnostic delay, ALB, LDH, WBC, HGB, LYMPH, MONO, and NEUT. After adjusting for age, sex, smoking, hypertension, diabetes, cerebrovascular disease, and cardiovascular disease, significance was retained for all eight factors, especially for ALB (OR=0.82, 95% CI: 0.75 to 0.91, *p* <0.001), LYMPH (OR=0.42, 95% CI: 0.27 to 0.64, *p* <0.001), and NEUT (OR=1.40, 95% CI: 1.16 to 1.70, *p* <0.001).

**Table 2:** Identification of significant factors in association with severe or critical COVID-19 relative to mild or ordinary COVID-19.

**Significant factors**	**Unadjusted**			Adjusted^*^		
	**OR**	**95% CI**	***p***	**OR**	**95% CI**	***p***
Diagnostic delay (+1)	1.08	1.01 to 1.16	0.036	1.08	1.02 to 1.17	0.048
ALB (+1)	0.84	0.77 to 0.91	<0.001	0.82	0.75 to 0.91	<0.001
LDH (+100)	1.61	1.15 to 2.27	0.006	1.56	1.14 to 2.13	0.011
WBC (+1)	1.24	1.06 to 1.46	0.007	1.27	1.08 to 1.50	0.004
HGB (+10)	0.68	0.53 to 0.88	0.004	0.68	0.52 to 0.90	0.008
LYMPH (+10)	0.42	0.28 to 0.64	<0.001	0.42	0.27 to 0.64	<0.001
MONO (+1)	0.85	0.75 to 0.97	0.019	0.85	0.75 to 0.98	0.019
NEUT (+1)	1.37	1.14 to 1.64	0.001	1.40	1.16 to 1.70	<0.001

Abbreviations: OR, odds ratio; 95% CI, 95% confidence interval; ALB, albumin; LDH, lactate dehydrogenase; WBC, white blood cell; HGB, hemoglobin; LYMPH, lymphocyte; MONO, monocyte; NEUT, neutrophil. The *p* value was calculated after adjusting for age, sex, smoking, hypertension, diabetes, cerebrovascular disease, and cardiovascular disease.

### Correlation analysis of significant factors for severe or critical COVID-19

Spearman correlation analysis was performed to examine the pairwise relationship between eight significant factors (data not shown). Due to the strong correlation of HGB, LYMPH, and MONO with the other factors, they were not kept in the following analyses.

### Prediction performance of five independent significant factors

**Figure 1:**
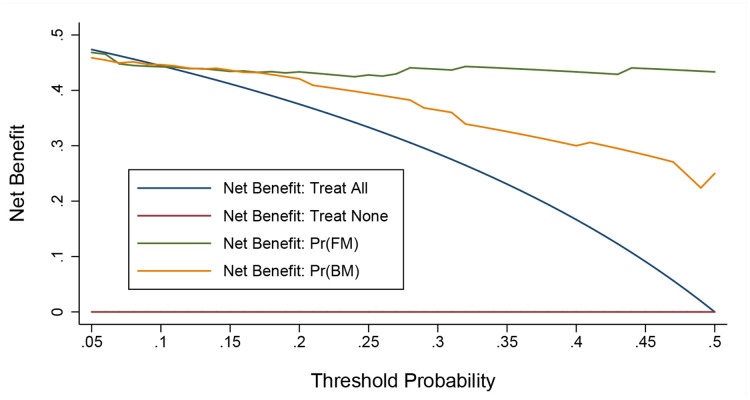
**Decision curve analysis on the net benefits gained by adding significant factors to the basic model.** Abbreviations: FM, full model; BM, basic model. The orange solid line corresponds to the basic model that includes age, sex, smoking, hypertension, diabetes, cardiovascular disease, and cerebrovascular disease. The green solid line corresponds to the full model that includes both factors in the basic model and the five newly-identified unrelated significant factors including diagnostic delay, albumin, lactate dehydrogenase, white blood cell, and neutrophil. Over threshold probabilities of 0.17, the net benefit gained by adding the five significant factors was greater than that in the basic model.

Before model construction on the basis of five independent significant factors, a wide range of statistics were calculated to assess prediction accuracy from both calibration and discrimination aspects (Table 3), as well as from the net benefits gained by adding the five factors to the basic model (Figure 1). Multi-aspect analyses revealed that the contribution of the five factors to predict the occurrence of severe or critical COVID-19 was statistically significant.

**Table 3:** Prediction performance for adding five significant factors to the basic model.

**Statistics**	Basic model*	**Basic model + significant factors**
AIC	112	60
BIC	131	93
LR test (*p*)		<0.0013
HL test (*p*)	0.325	0.186
NRI (*p*)		0.001
IDI (*p*)		<0.001
AUROC	0.86 (0.76, 0.95)	0.96 (0.91, 1.00)
AUROC (*p*)		0.026

Abbreviations: AIC, Akaike information criterion; BIC, Bayesian information criterion; LR, likelihood ratio; HL test, Hosmer-Lemeshow test; NRI, net reclassification improvement; IDI, integrated discrimination improvement; AUROC, area under the receiver operating characteristic. *Variables in the basic model included age, sex, smoking, hypertension, diabetes, cardiovascular disease, and cerebrovascular disease.

### Establishment of prediction nomogram model

**Figure 2:**
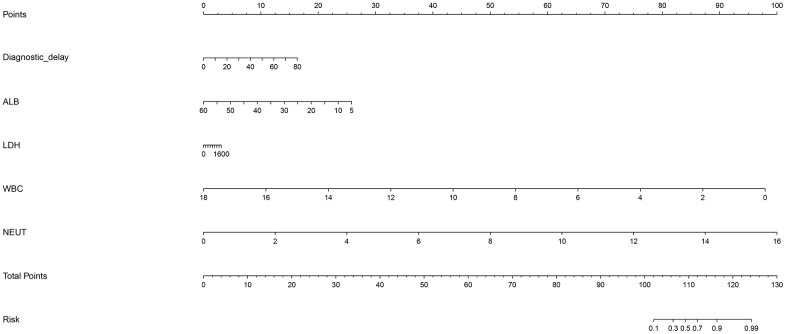
**Prediction nomogram for the prediction of severe or critical COVID-19 relative to mild or ordinary COVID-19 based on the five newly-identified unrelated significant factors.** Abbreviations: ALB, albumin; WBC, white blood cell; NEUT, neutrophil. This nomogram can be used to manually obtain predicted values from a regression model that was fitted with the five significant factors including diagnostic delay, albumin, lactate dehydrogenase, white blood cell, and neutrophil. In detail, there is a reference line at the top for reading scoring points (range: 0 to 100) from all factors in the regression model, which were summed together to calculate the total points, and then the predicted values can be read at the bottom.

To further analyze the joint contribution of five independent significant factors, a prediction nomogram model was established, as shown in Figure 2. The maximal prediction capability reached as high as 99%, and calibration curve showed good prediction performance (Supplementary Figure 1), as reflected by the C-index (90.6%, *p* <0.001).

## DISCUSSION

Via a cross-sectional analysis of 370 hospitalized patients with COVID-19 at a tertiary hospital, Yichang city, in the same province where Wuhan city is located, we have identified five independent factors in significant association with severe or critical COVID-19 relative to mild or ordinary COVID-19, and their prediction was reinforced in a nomogram model. The findings of this study can help enrich our understanding on the risk profiles for the progression of COVID-19 from mild or ordinary symptoms to severe or critical symptoms.

Waves of studies that have attempted to identify factors such as demographic characteristics, medical histories, laboratory biomarkers responsible for the onset and progression of COVID-19 symptoms are coming toward us like a tsunami since early 2020 [11–20]. Currently, one of the pressing problems facing clinicians is that the significant factors identified by individual studies are not often reproducible. Besides inadequate statistical power due to small sample sizes, many times, such irreproducibility may be attributed to the failure to adequately adjust for confounding. Several techniques are recommended to control or reduce the impact of confounding factors, such as statistical adjustment, subsidiary exploration, and propensity score matching. Growing evidence indicates that multiple reports have shown underlying chronic health conditions such as hypertension, diabetes, cardiovascular disease, and cerebrovascular disease are overrepresented and tend to be associated with severe COVID-19 [4, 21–24], in line with the findings of the present cross-sectional analysis. To account for these established underlying chronic health conditions, we here employed the propensity score matching method to balance baseline covariates between patients with mild or ordinary COVID-19 and severe or critical COVID-19 in order to “replicate” a randomized controlled trial. Propensity score matching is a tool for causal inference in non-randomized studies that allows for conditioning on large sets of covariates [25].

After propensity score matching and statistical adjustment, we identified five uncorrelated factors that were independently and statistically associated with the risk of having severe or critical COVID-19 relative to mild or ordinary COVID-19, including diagnostic delay, ALB, LDH, WBC, and NEUT, consistent with the findings of some recent studies [26–33]. Given the fact that clinical progression of COVID-19 symptoms is a multistep, multifactorial progress, it is unlikely that any one single factor would play a predominant part in this process. There is a wide recognition that a risk prediction model regressing multiple attributes is more imperative than reporting single significant attributes alone, because the contribution of a single attribute may be enhanced or shadowed by the concurrent presence of another attribute. To shed some light on this issue, we attempted to establish a prediction nomogram model on the basis of factors that were in week correlation, independent of demographic covariates, in significant association with severe or critical COVID-19, and exhibited decent prediction performance from multiple aspects. As expected, this nomogram model had good prediction capability and accuracy, albeit our analysis was based on mere 30% of original data after propensity score matching. In the literature, several studies have attempted to predict the severity and mortality of COVID-19 by using the nomogram technique [34–37], yet the factors modelled in the nomogram were not consistent studies, possibly due to differences in patient characteristics and statistical power, as well as possible residual confounding. As such, we expect further external validation of our significant findings in other independent populations.

Several limitations should be acknowledged for this study. Firstly, all study patients with COVID-19 were consecutively enrolled from a mono-center, and it could better be generalized pending consistently confirmed in other cohorts. Second, this study was designed in a cross-sectional pattern, and making references regarding causality is not allowed. Thirdly, assessable patients with COVID-19, especially with severe or critical symptoms are less than 50, which prohibits further subsidiary analyses to explore confounding effects. Fourthly, our findings were exclusively derived from a Chinese population, and external validation in other racial populations would be of added interest.

Taken together, our findings provide evidence for the significant contribution of five independent factors to the risk of severe or critical COVID-19 relative to mild or ordinary COVID-19. More importantly, a prediction nomogram model based on the five factors is useful for the identification of high-risk patients with mild or ordinary COVID-19 in predisposition to severe or critical symptoms.

## MATERIALS AND METHODS

### Study patients

All study patients, who were confirmed to be infected with COVID-19, were consecutively enrolled at The Third People’s Hospital of Yichang, Yichang city, Hubei province, China during the period from January to March 2020. They received medical treatment and/or standard care for COVID-19 after infection in this hospital. All patients willingly gave written informed consent for participation after a full explanation of the procedures associated with this study. No restrictions were imposed with regard to the age, gender, and ethnicity or COVID-19 severity of affected patients under study.

### Diagnosis of COVID-19

COVID-19 was diagnosed according to the coronavirus disease 2019 diagnosis and treatment plan (tentative sixth edition) released by the National Health and Health Committee of China [38, 39]. The confirmation of COVID-19 was made by the 2019-Novel Coronavirus (2019-nCoV) Real-time PCR Kit.

### Severity criteria of COVID-19

According to the coronavirus disease 2019 diagnosis and treatment plan (tentative sixth edition) released by the National Health and Health Committee of China [38, 39], patients with COVID-19 can be classified into four clinical types, that is, mild cases, ordinary cases, severe cases, and critical cases. Mild cases refer to mild clinical symptoms and no detectable pneumonia manifestation in imaging. Ordinary cases include patients who have symptoms like fever and respiratory tract symptoms and detectable pneumonia manifestation in imaging. Severe cases are recorded if any of the following items is met: (a) respiratory distress, respiratory rate ≥ 30 breaths/min; (b) pulse oxygen saturation (SpO2) ≤93% on room air at rest state; (c) arterial partial pressure of oxygen (PaO2)/fraction of inspired oxygen (FiO2) ≤300 mmHg. At higher altitudes (above 1 km), PaO2/FiO2 values should be adjusted based on equation of PaO2/FiO2 × [Atmospheric Pressure (mmHg)/760]; (d) patients with >50% lesions progression within 24 to 48 h in pulmonary imaging. Critical cases are recorded if any of the following items is met: (a) respiratory failure occurs and mechanical ventilation is required; (b) shock occurs; (c) complicated with other organ failure that requires monitoring and treatment in Intensive Care Unit (ICU).

### Demographic information

After informed written consent, all eligible patients were interviewed and demographic information was recorded, including age at admission, gender, contact with Wuhan citizens, cigarette smoking, hypertension, diabetes, cerebrovascular disease, cardiovascular disease, and diagnostic delay (days). Cigarette smoking is grouped into never smoking and ever smoking, and ever smoking includes former and current smoking. Hypertension is defined as systolic blood pressure (SBP) of ≥140 mm Hg, diastolic blood pressure (DBP) of ≥90 mm Hg, or current use of antihypertensive medicine. Diabetes is defined as fasting plasma glucose concentration ≥7.0 mmol/L or a self-reported diagnosis. Diagnostic delay refers to the time between onset of symptoms and first diagnosis of COVID-19, and is recorded in days.

### Laboratory biomarkers

Laboratory biomarkers were measured at the time of enrollment, including white blood cell (WBC), neutrophil (NEUT), lymphocyte (LYMPH), monocyte (MONO), platelet (PLT), procalcitonin (PCT), total bilirubin (TBIL), direct bilirubin (DBIL), albumin (ALB), alanine transaminase (ALT), aspartate aminotransferase (AST), creatinine (Cr), creatine kinase (CK), creatine phosphokinase-isoenzyme-MB (CKMB), C-reactive protein (CRP), lactate dehydrogenase (LDH), and hemoglobin (HGB). All biomarkers were assayed at the Laboratory Department of The Third People’s Hospital of Yichang.

### Statistical analyses

Continuous data are summarized as median (interquartile range), and categorical data are summarized as count (percentage). Two-group comparison was done by using the Wilcoxon rank-sum test or χ^2^ test, when appropriate. Propensity score matching method was used to balance confounding factors, and it was implemented by using the order “psmatch2” in the STATA software (v14.1). Logistic regression analysis was used to identify potential risk factors for severe or critical COVID-19 relative to mild or ordinary COVID-19 before and after adjusting for confounding factors. Prediction accuracy was statistically assessed using calibration and discrimination statistics, including Akaike information criterion (AIC), Bayesian information criterion (BIC), likelihood ratio (LR) test, Hosmer-Lemeshow (HL) test, net reclassification improvement (NRI), integrated discrimination improvement (IDI), and area under the receiver operating characteristic (AUROC), as well as visually by decision curve analysis (DCA). Finally, based on the significant factors, a prediction nomogram model was established in prediction of severe or critical COVID-19. In addition, a calibration curve was plotted and C-index was estimated to assess prediction performance. Nomogram, calibration curve, and C-index were implemented by the “RMS” package (v3.6.3) in the R software (v3.6.1). The STATA software (v14.1) was used for statistical analyses unless otherwise illustrated.

### Ethical approval

The conduct of this study was reviewed and approved by both Ethics Committees of The Third People’s Hospital of Yichang and The First Affiliated Hospital of Fujian Medical University (Approval No. MRCTA, ECFAH of FMU 2020-153).

### Data availability statement

Data involved in this study are available upon reasonable request.

## Supplementary Material

Supplementary Figure 1

Supplementary Table 1
