# Supplementary material for: Predicting severe or critical symptoms in hospitalized patients with COVID-19 from Yichang, China
Source: Aging (Albany NY). 2020 Dec 9;13(2):1608–19. doi: 10.18632/aging.202261 (PMC7880337; doi:10.18632/aging.202261)
Supplement: Supplementary Figure 1 [file aging-13-202261-s001.pdf]

SUPPLEMENTARY FIGURE

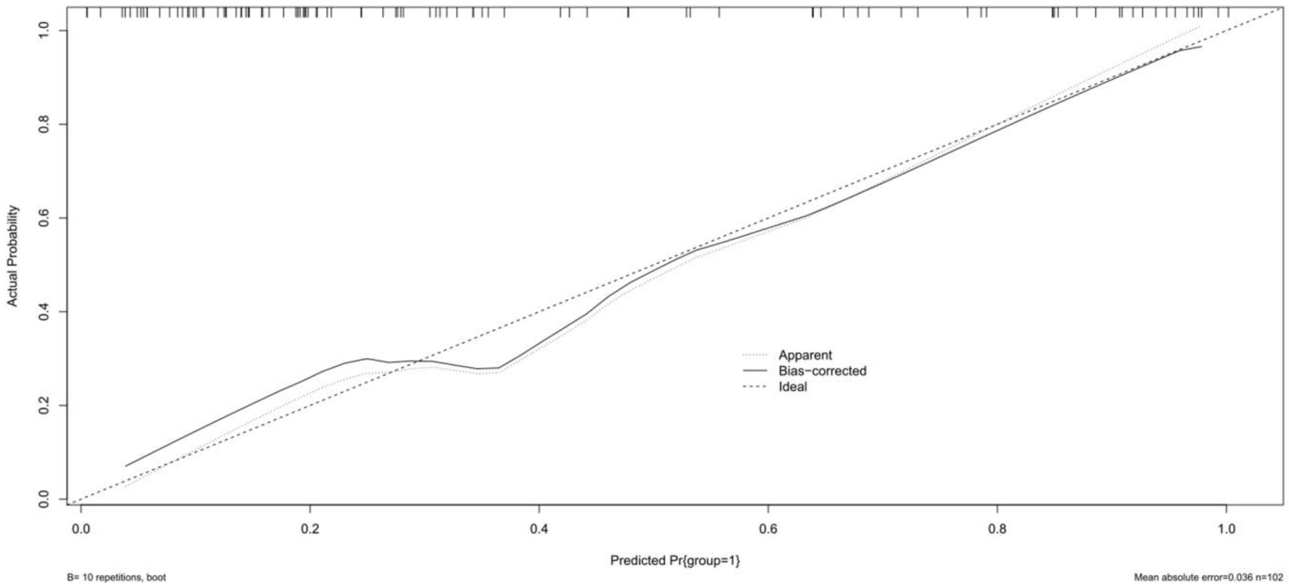

Supplementary Figure 1. Calibration curve of the nomogram prediction model for severe or critical COVID-19 relative to mild or ordinary COVID-19.
