# Supplementary material for: Predicting severe or critical symptoms in hospitalized patients with COVID-19 from Yichang, China
Source: Aging (Albany NY). 2020 Dec 9;13(2):1608–19. doi: 10.18632/aging.202261 (PMC7880337; doi:10.18632/aging.202261)
Supplement: Supplementary Table 1 [file aging-13-202261-s002.pdf]

Supplementary Table

Supplementary Table 1. The baseline characteristics of study patients with COVID-19.

Characteristics	Mild or ordinary cases (n=326)	Severe or critical cases (n=44)	*p*
Age (years)	51 (38, 65)	70 (60, 79)	<0.001
Males	176 (54%)	24 (54.5%)	0.944
Contact with WH	127 (41.8%)	40 (90.9%)	<0.001
Death	0 (0%)	16 (36.4%)	<0.001
Hypertension	63 (19.4%)	22 (50%)	<0.001
Diabetes	31 (9.5%)	11 (25%)	0.002
Smoking	50 (15.4%)	6 (13.6%)	0.762
Cerebrovascular disease	6 (1.8%)	5 (11.4%)	<0.001
Cardiovascular disease	10 (3.1%)	9 (20.5%)	<0.001
Diagnostic delay (days)	3 (2, 5)	6 (3, 10)	<0.001
WBC	4.400 (3.400, 5.595)	5.600 (4.450, 7.800)	<0.001
NEUT	61.70 (53.80, 69.50)	81.10 (67.80, 89.70)	<0.001
LYMPH	27.95 (21.60, 35.10)	12.85 (6.500, 22.65)	<0.001
MONO	7.100 (5.200, 9.200)	4.250 (2.600, 7.200)	<0.001
NEUT (×109/L)	2.640 (1.900, 3.660)	4.635 (2.940, 6.690)	<0.001
LYMPH (×109/L)	1.205 (0.870, 1.550)	0.755 (0.515, 1.015)	<0.001
MONO (×109/L)	0.300 (0.230, 0.400)	0.280 (0.200, 0.365)	0.159
PLT (×109/L)	139 (110, 190)	136 (92, 181.5)	0.121
PCT (ng/mL)	0.0800 (0.0500, 0.120)	0.135 (0.0800, 0.265)	<0.001
TBIL (μmol/L)	9.010 (6.660, 13.03)	9.030 (6.935, 14.55)	0.611
DBIL (μmol/L)	2.310 (1.550, 3.420)	3.100 (2.250, 4.970)	<0.001
ALB (g/L)	38.15 (34.75, 41.15)	29.60 (27.40, 36.10)	<0.001
ALT (UL)	21 (14, 33)	23.50 (14.50, 34.50)	0.695
AST (UL)	21 (16, 28)	24 (21, 36.50)	0.006
Cr (μmol/L)	68.20 (54.60, 79.60)	65.60 (54.55, 83.95)	0.777
CK (UL)	59 (40, 99)	84 (46, 132)	0.144
CKMB (UL)	11.80 (9.400, 16)	15.05 (9.900, 27.10)	0.021
CRP (mg/L)	17.20 (4, 43.30)	42.95 (13.35, 69)	<0.001
LDH (UL)	202.5 (163.5, 258.5)	315.5 (217, 454)	<0.001
HGB (g/L)	122 (111, 134)	114 (98, 122.5)	<0.001

Abbreviations: WH, Wuhan; WBC, white blood cell; NEUT, neutrophil; LYMPH, lymphocyte; MONO, monocyte; PLT, platelet; PCT, procalcitonin; TBIL, total bilirubin; DBIL, direct bilirubin; ALB, albumin; ALT, alanine transaminase; AST, aspartate aminotransferase; Cr, creatinine; CK, creatine kinase; CKMB, creatine phosphokinase-isoenzyme-MB; CRP, C-reactive protein; LDH, lactate dehydrogenase; HGB, hemoglobin. The *p* value was calculated using the rank sum test or χ^2 test where appropriate. Data are expressed as median (interquartile range) or count (percentage).
